# Supplementary material for: FAT10 inhibits TRIM21 to down-regulate antiviral type-I interferon secretion
Source: Life Sci Alliance. 2024 Jul 8;7(9):e202402786. doi: 10.26508/lsa.202402786 (PMC11231494; doi:10.26508/lsa.202402786)
Supplement: Supplementary file 1 [file LSA-2024-02786_SdataF1_F2_F3_F4_F5_FS1_FS4_FS5.pdf]

Figure 1A

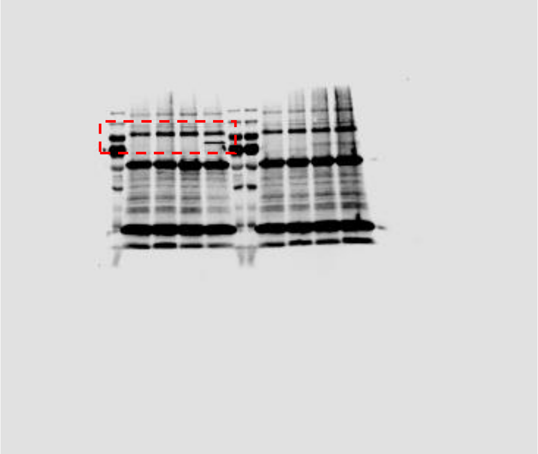

IP: FLAG
IB: HA

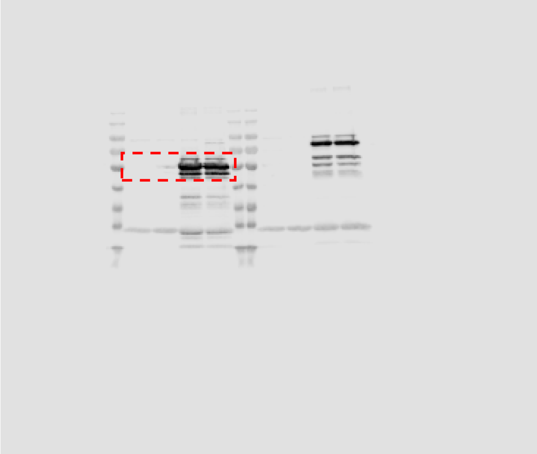

IP: FLAG
IB: FLAG

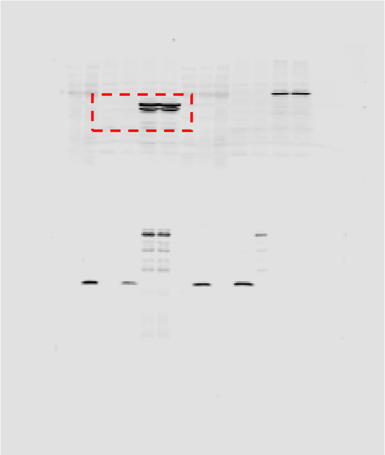

IB: FLAG
Load

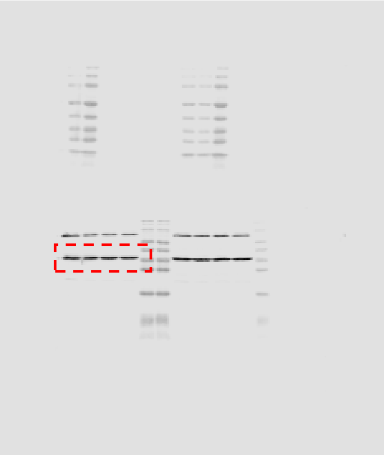

IB: GAPDH
Load

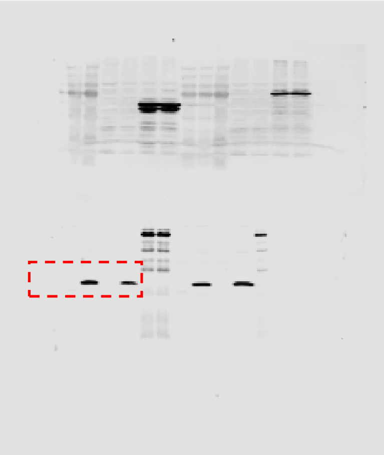

IB: HA
Load

Figure 1B

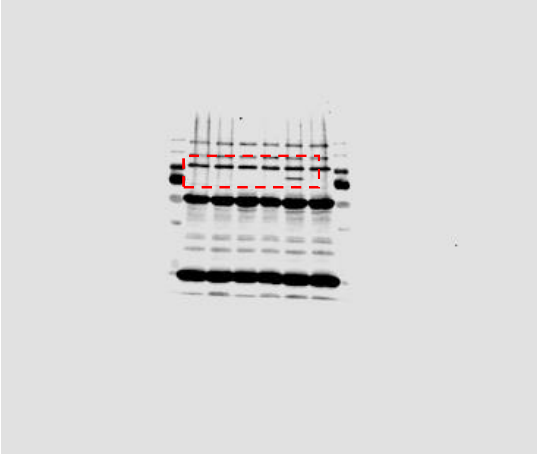

IP: FLAG
IB: HA

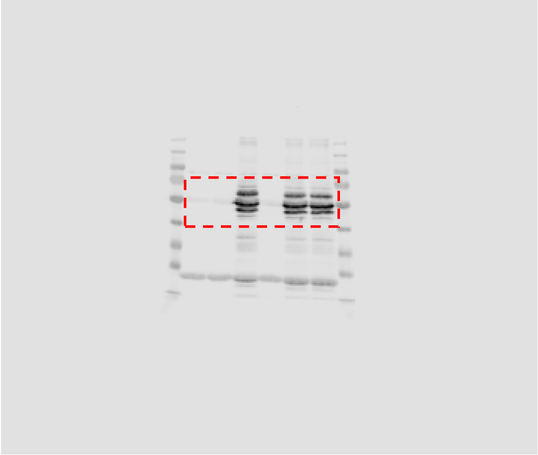

IP: FLAG
IB: FLAG

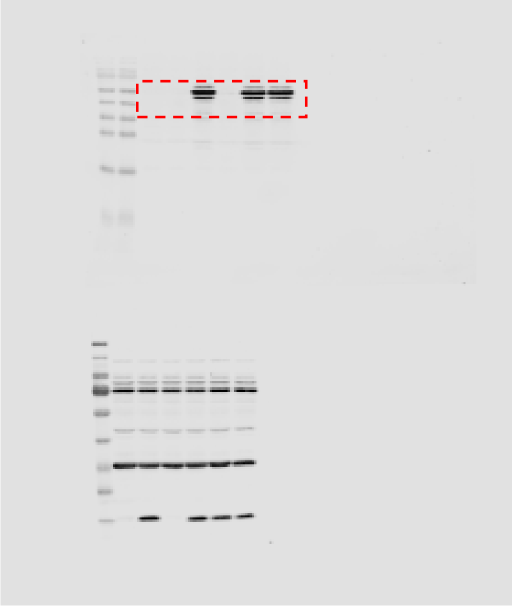

IB: FLAG
Load

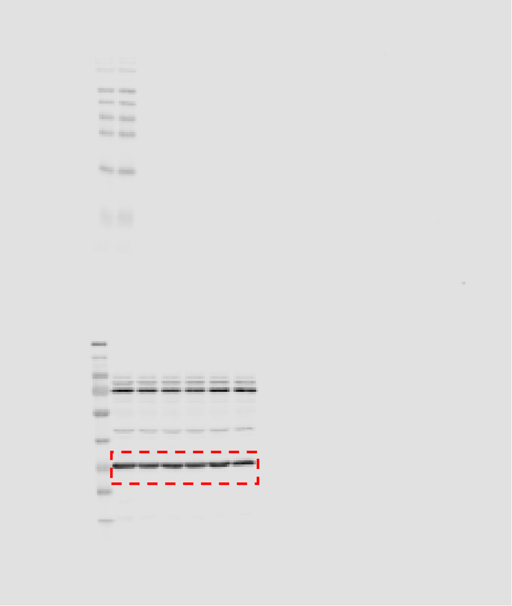

IB: GAPDH
Load

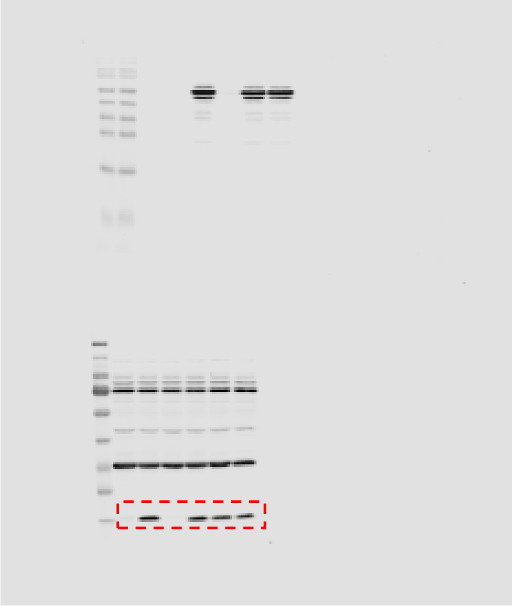

IB: HA
Load

Figure 1C

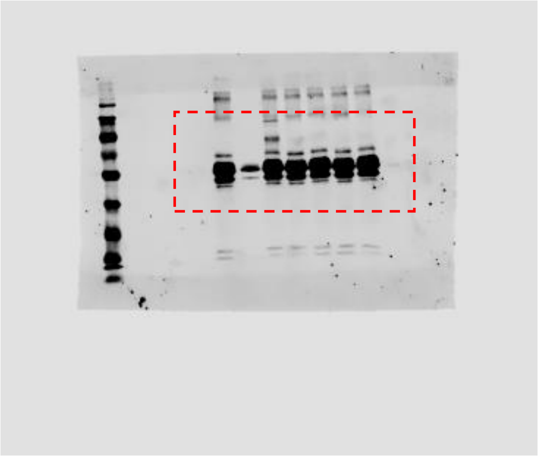

IB: TRIM21

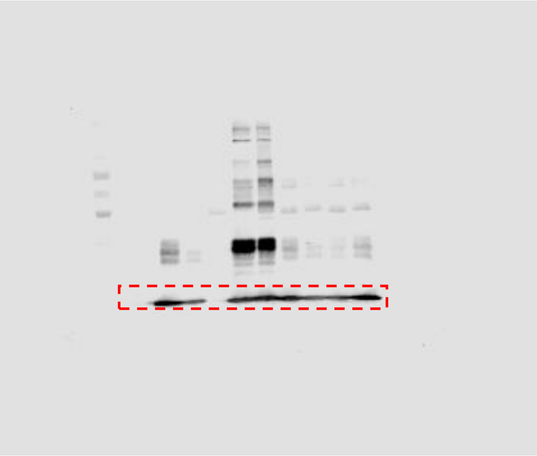

IB: FAT10

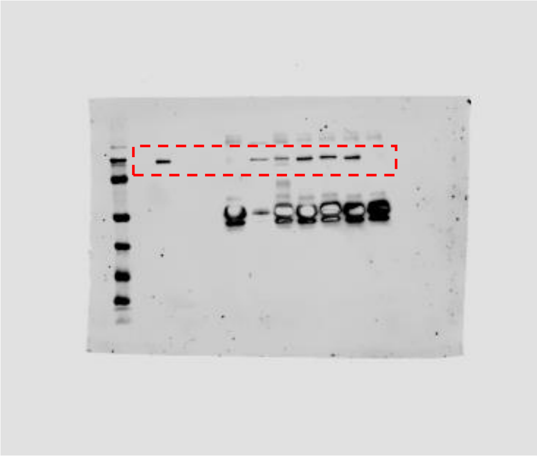

IB: UBA6

Figure 1E

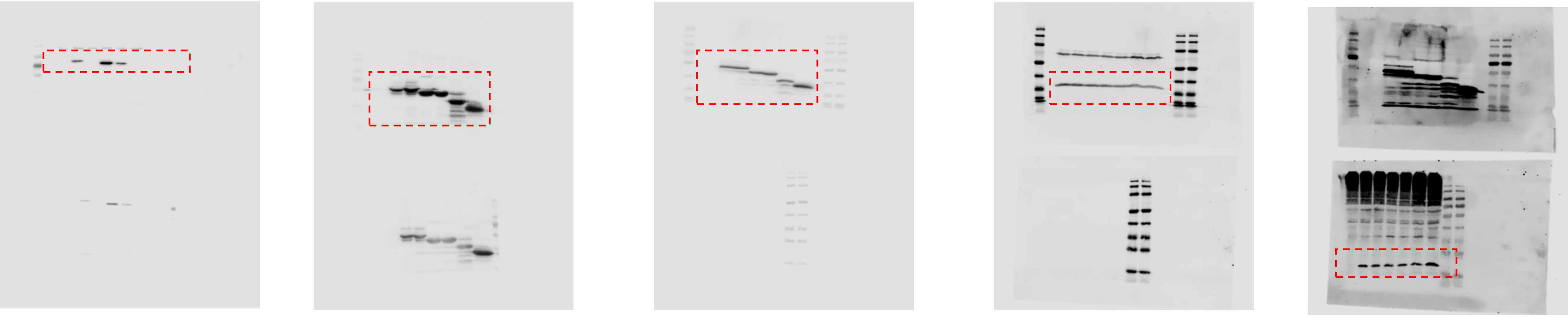

IP: FLAG
IB: HA

IP: FLAG
IB: FLAG

IB: FLAG
Load

IB: GAPDH
Load

IB: HA
Load

Figure 2A

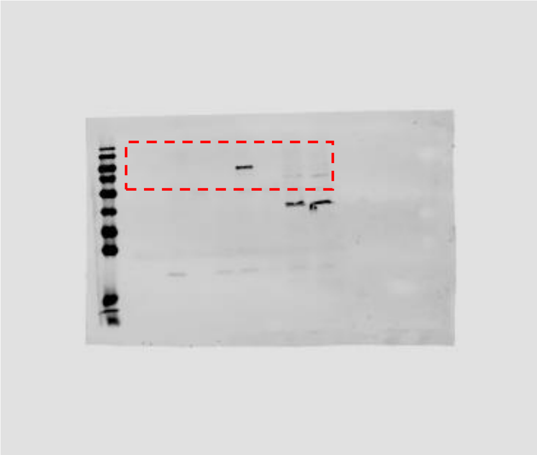

IP: FLAG
IB: HA

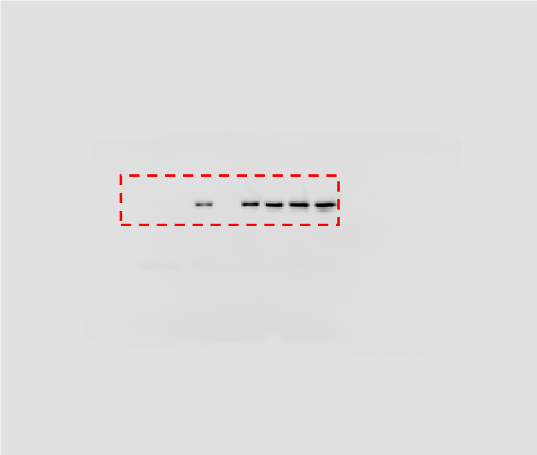

IP: FLAG
IB: FLAG

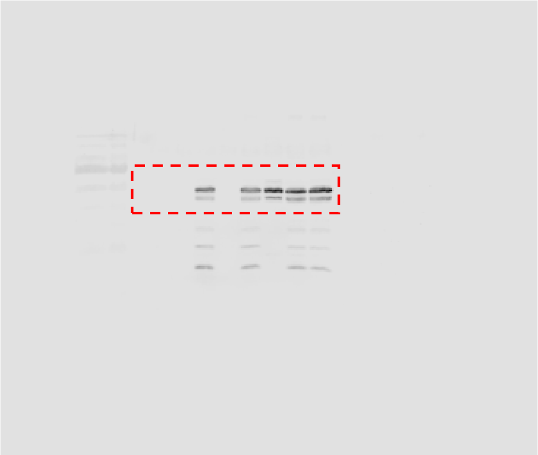

IB: FLAG
Load

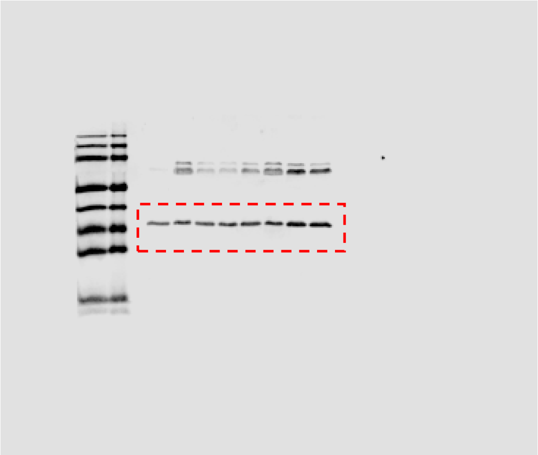

IB: GAPDH
Load

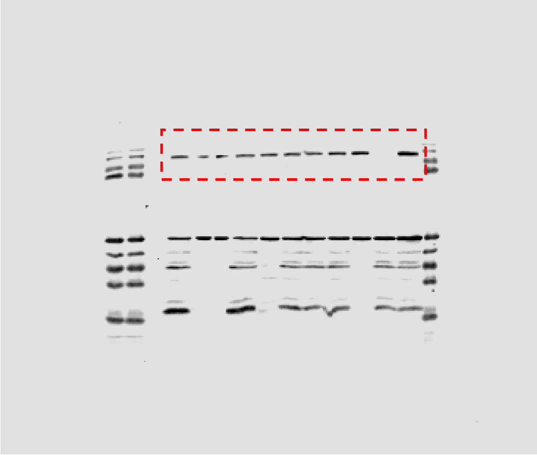

IB: UBA6
Load

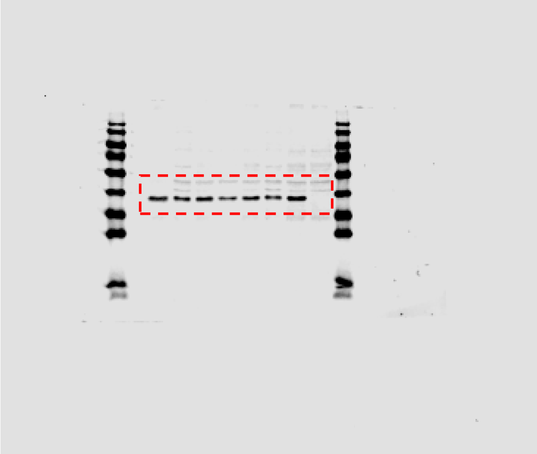

IB: USE1
Load

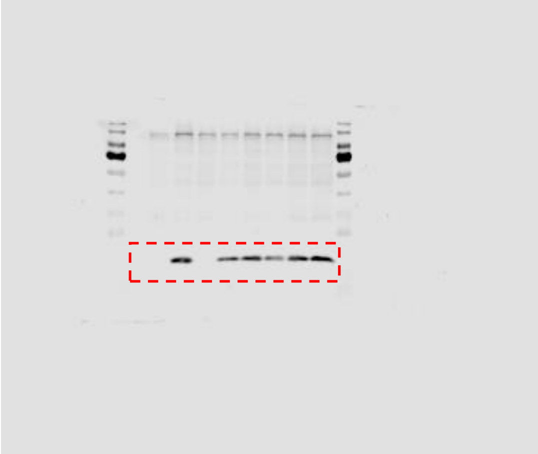

IB: HA
Load

Figure 2B

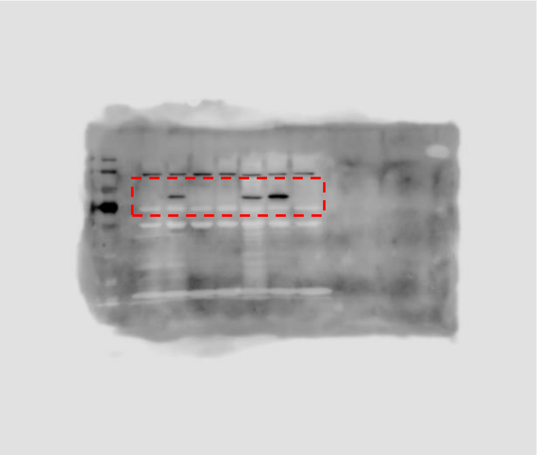

IP: FLAG
IB: HA

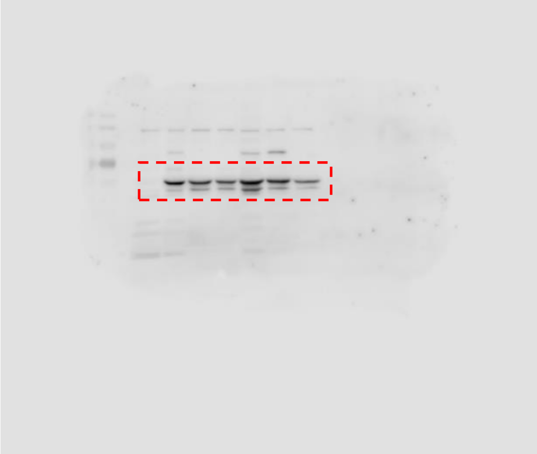

IP: FLAG
IB: FLAG

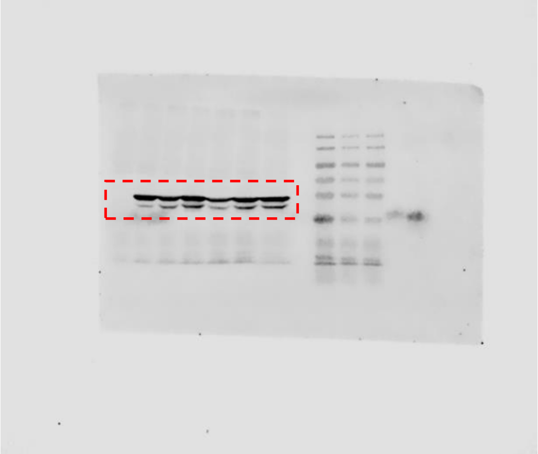

IB: FLAG
Load

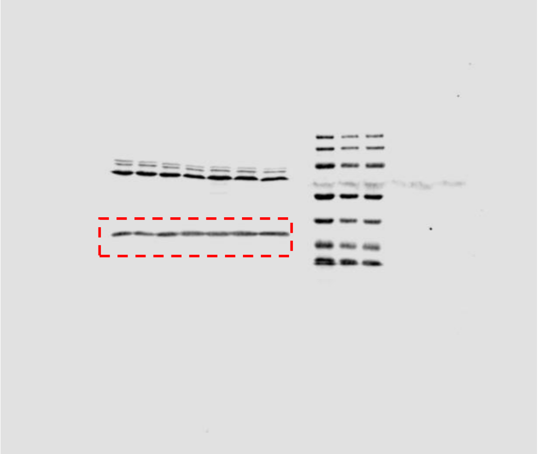

IB: GAPDH
Load

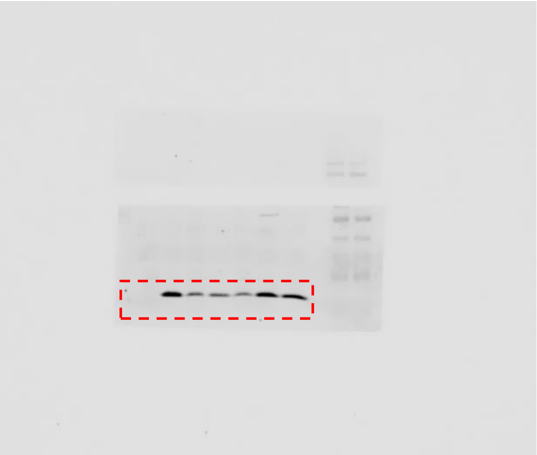

IB: HA
Load

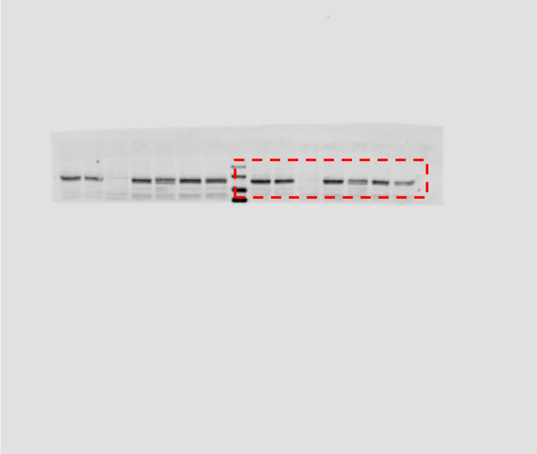

IB: UBA6
Load

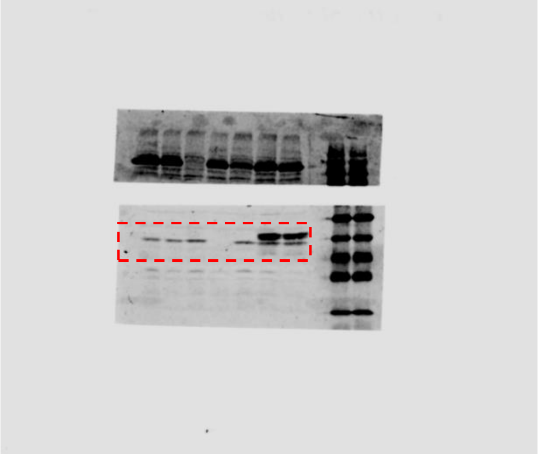

IB: USE1
Load

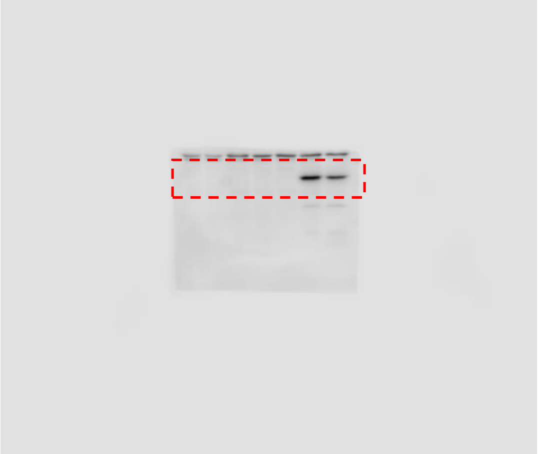

IB: HIS
Load

Figure 2C

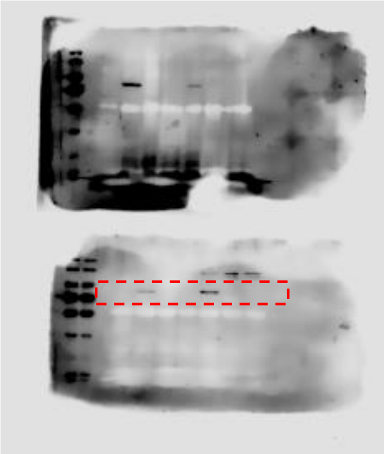

IP: FLAG
IB: HA

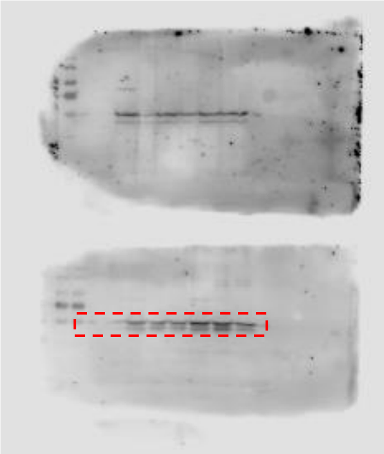

IP: FLAG
IB: FLAG

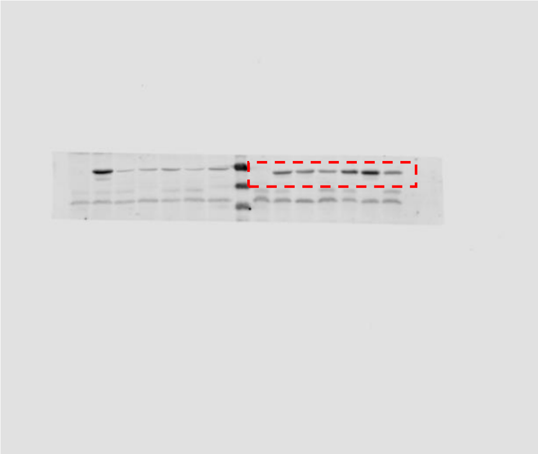

IB: FLAG
Load

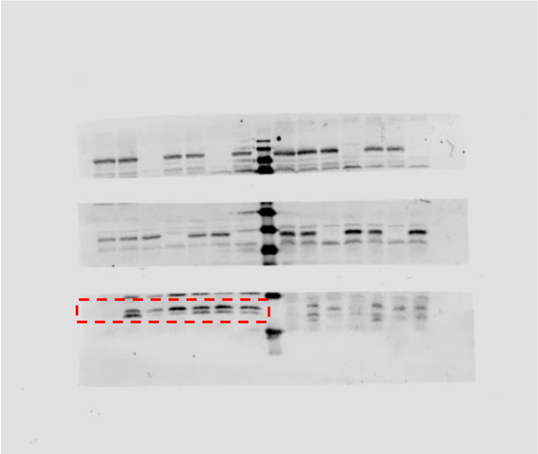

IB: HA
Load

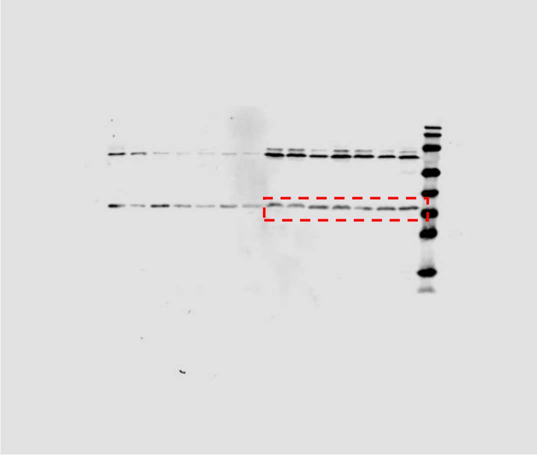

IB: GAPDH
Load

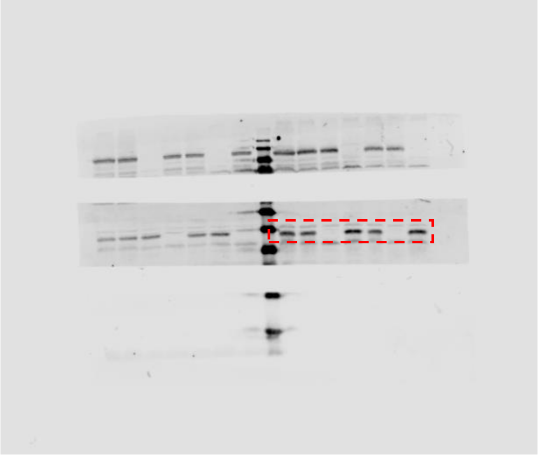

IB: UBA6
Load

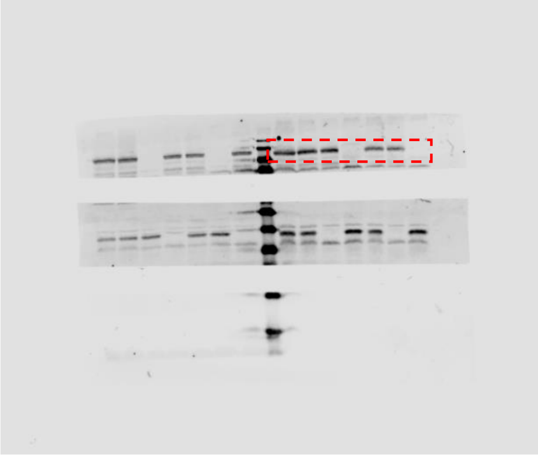

IB: USE1
Load

Figure 3A

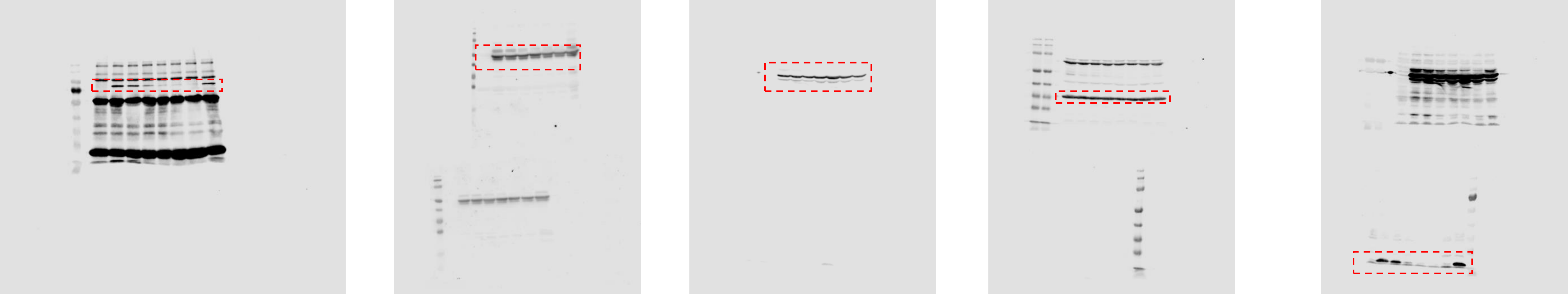

IP: FLAG
IB: HA

IP: FLAG
IB: FLAG

IB: FLAG
Load

IB: GAPDH
Load

IB: HA
Load

Figure 4A

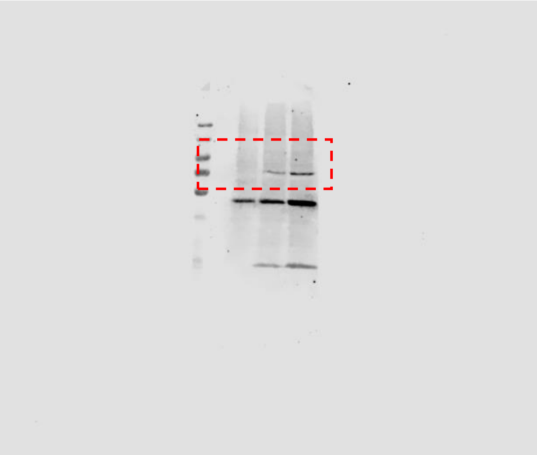

PD: Ni-IDA
IB: TRIM21

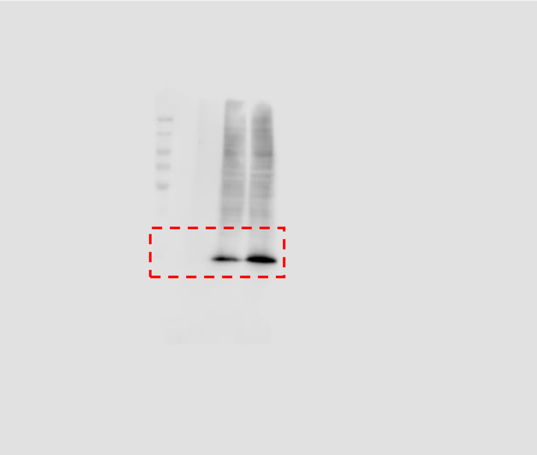

PD: Ni-IDA
IB: FLAG

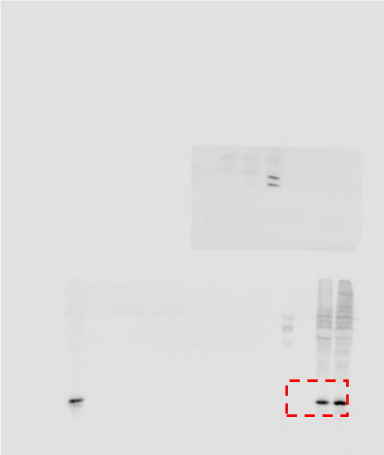

IB: FLAG
Load

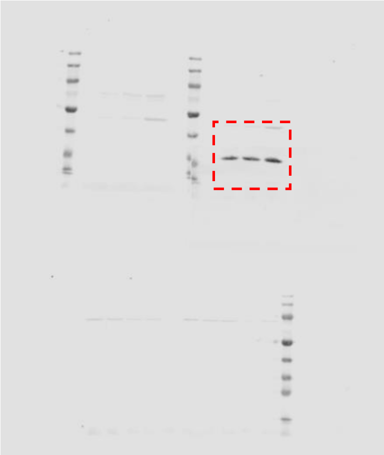

IB: GAPDH
Load

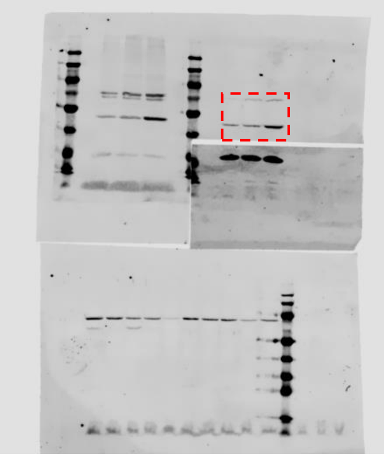

IB: TRIM21
Load

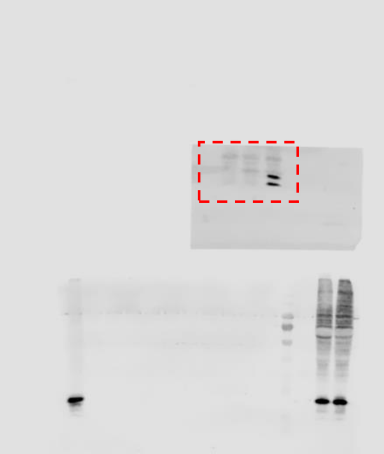

IB: M1
Load

Figure 4B

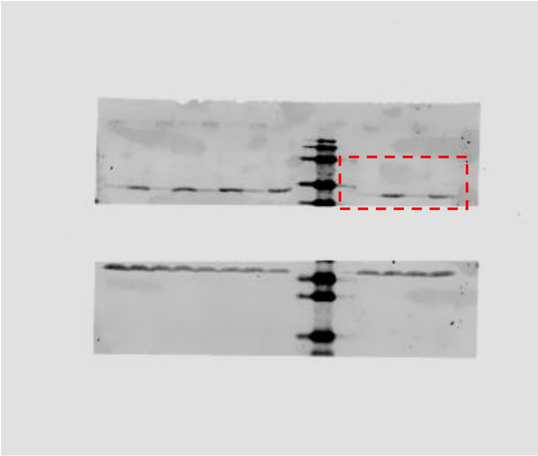

IB: TRIM21

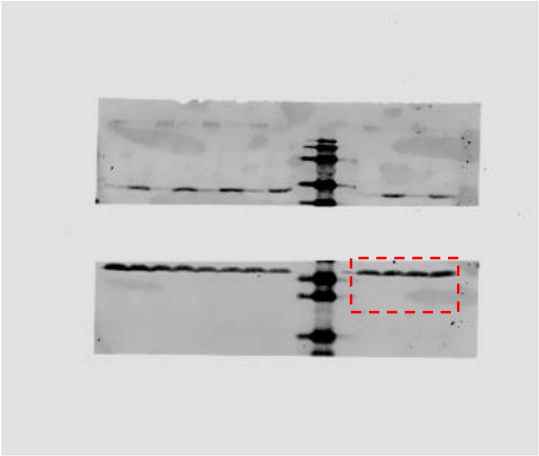

IB: GAPDH

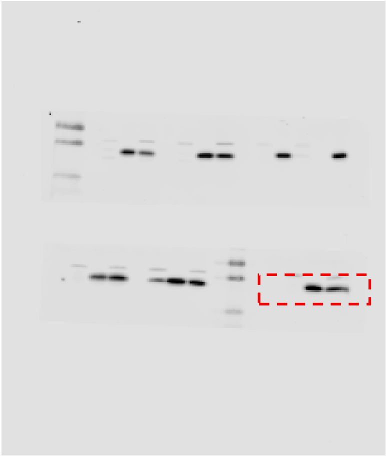

IB: FLAG

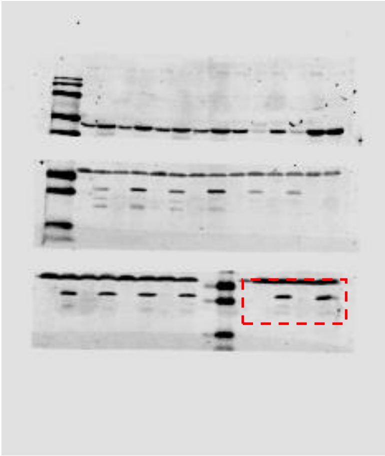

IB: M1

Figure 4D

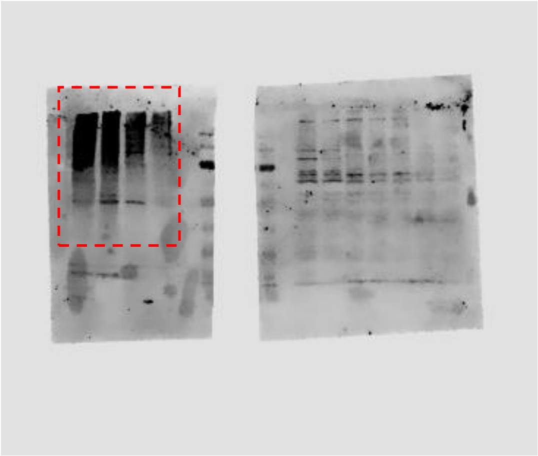

IP: TRIM21
IB: Ub

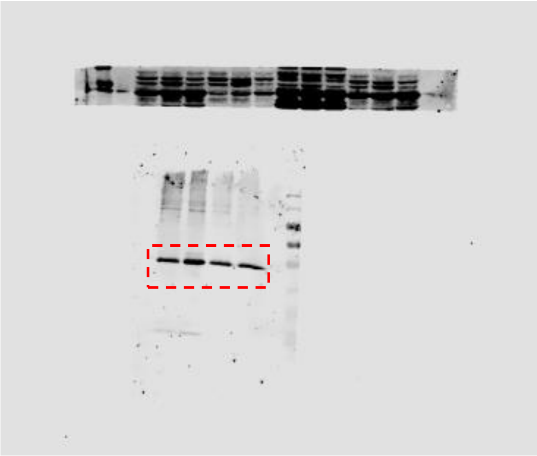

IP: TRIM21
IB: TRIM21

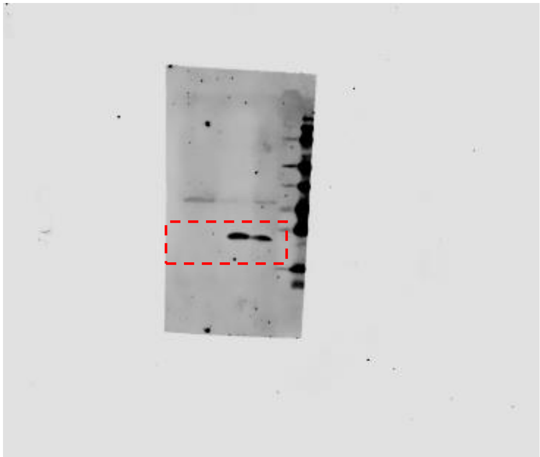

IB: FLAG
Load

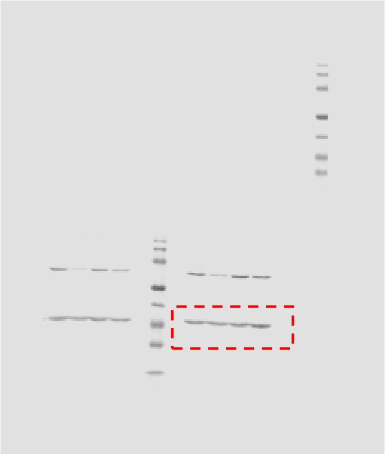

IB: GAPDH
Load

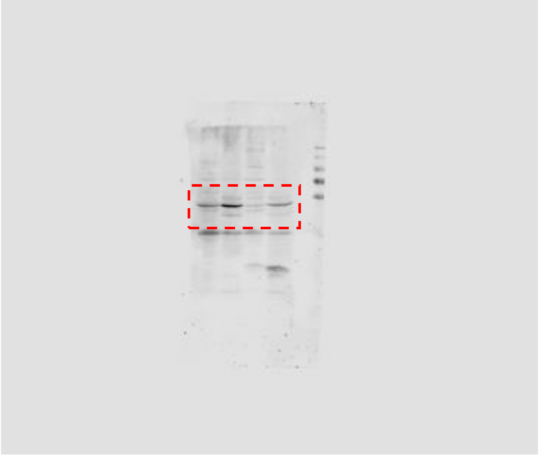

IB: TRIM21
Load

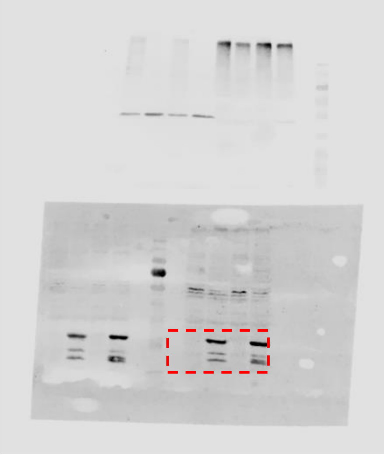

IB: M1
Load

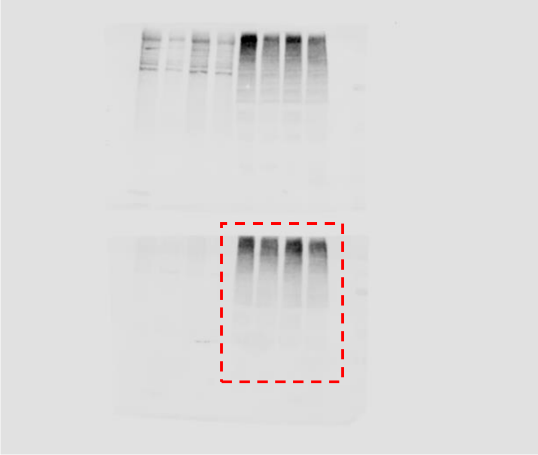

IB: Ub
Load

Figure 5A

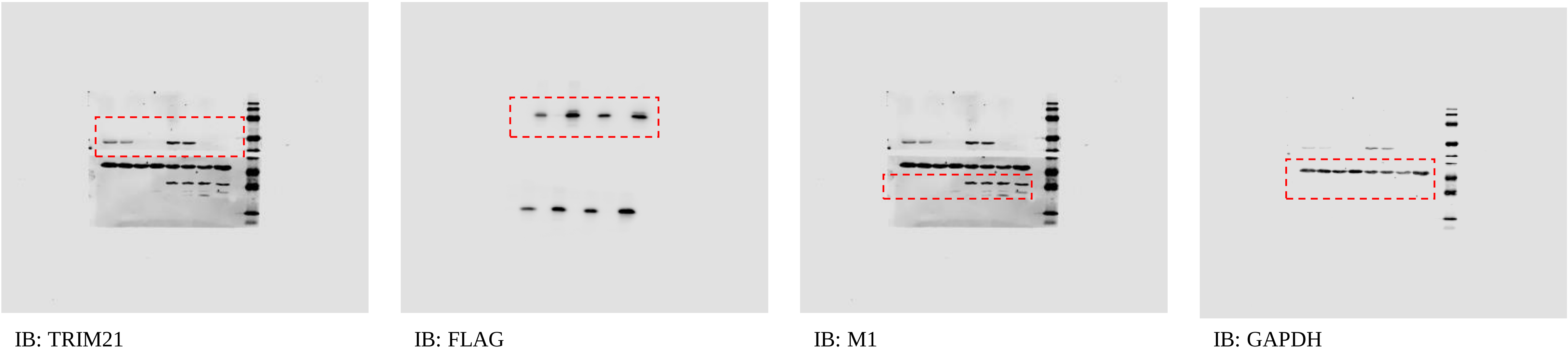

Figure 5C

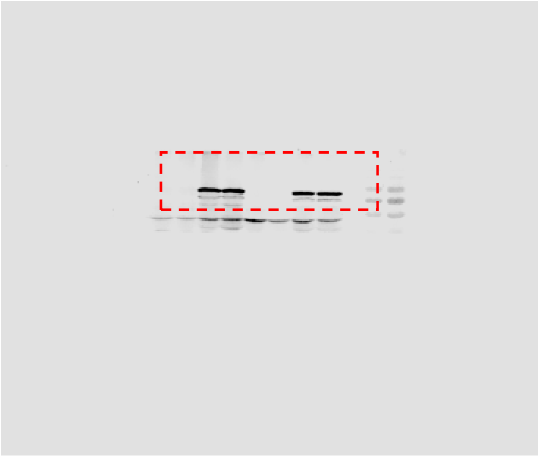

IB: TRIM21

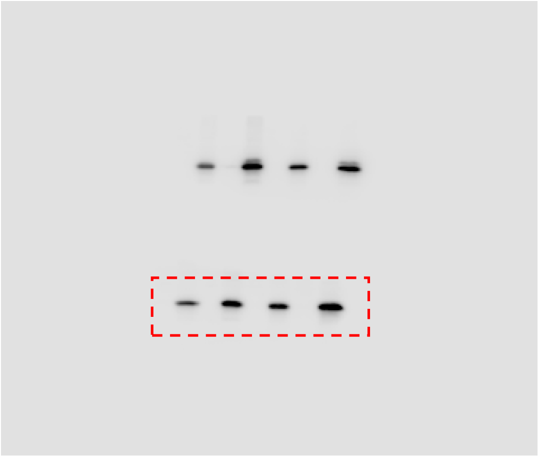

IB: FLAG

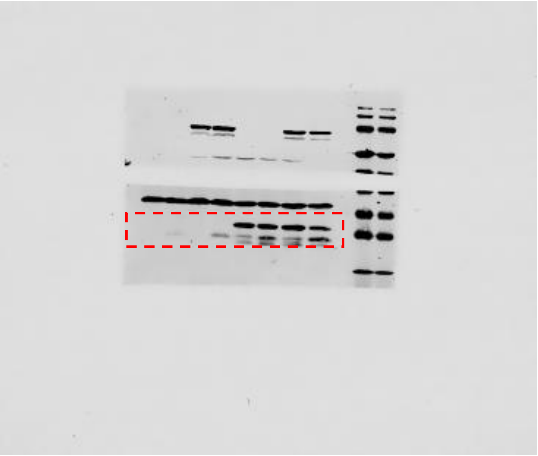

IB: M1

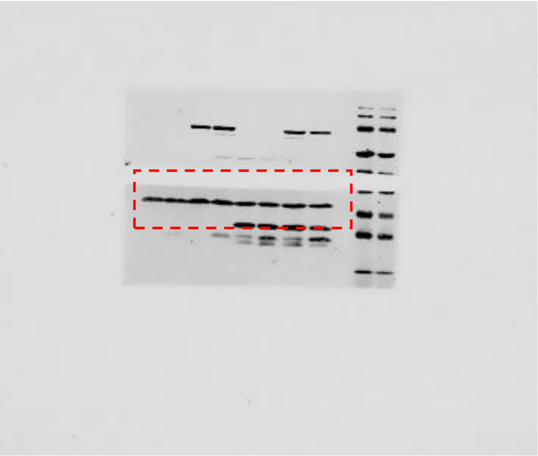

IB: GAPDH

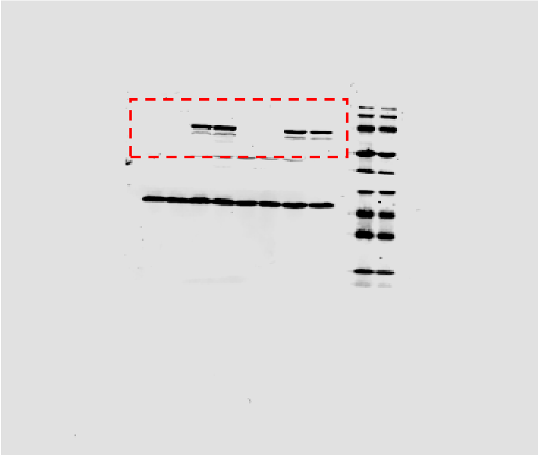

IB: mCherry

Figure S1A

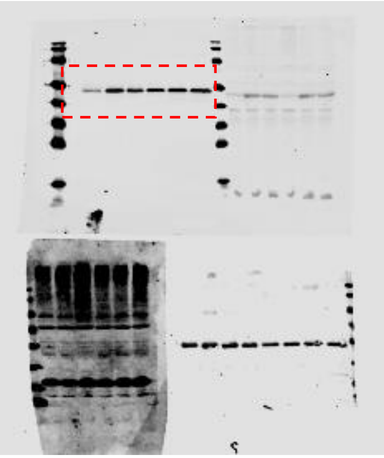

IP: TRIM21
IB: TRIM21

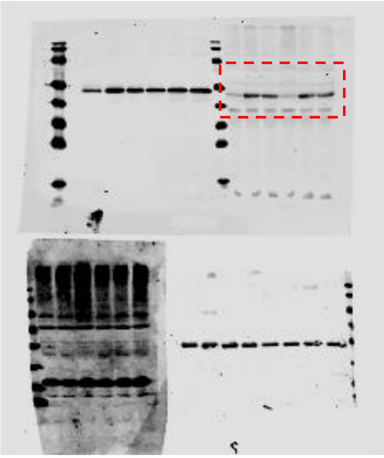

IB: TRIM21
Load

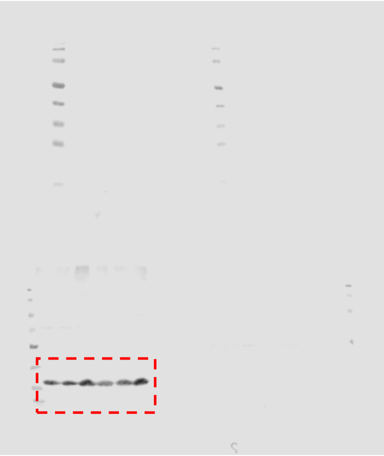

IB: GAPDH
Load

Figure S1B

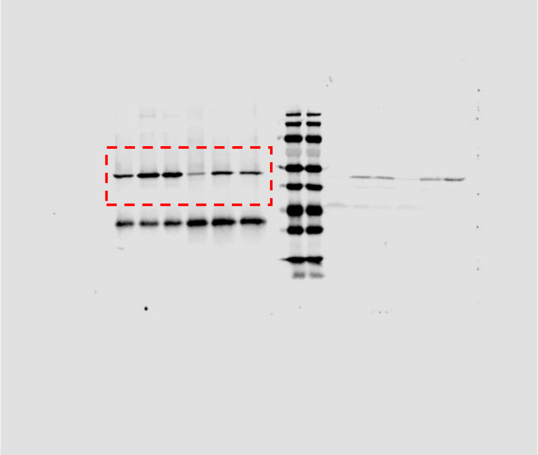

IP: FAT10
IB: TRIM21

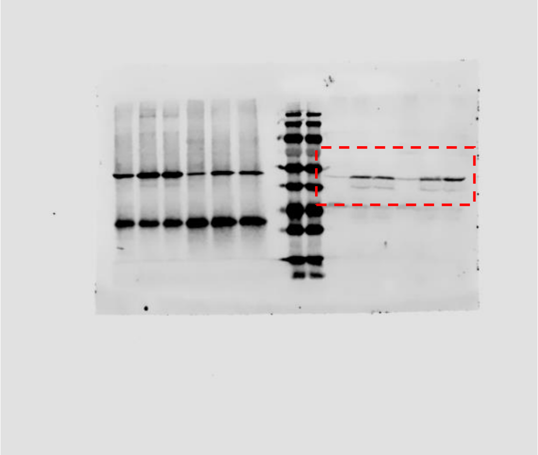

IB: TRIM21
Load

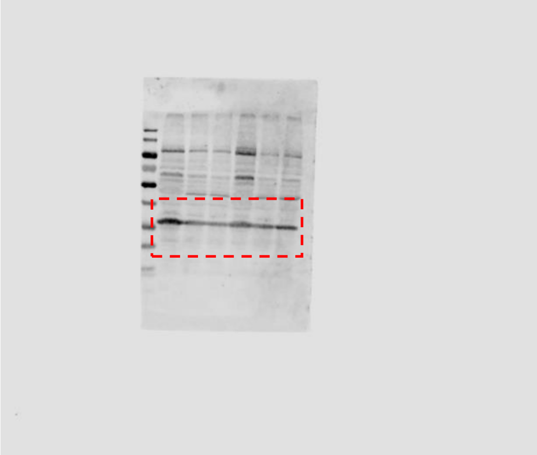

IB: GAPDH
Load

Figure S1C

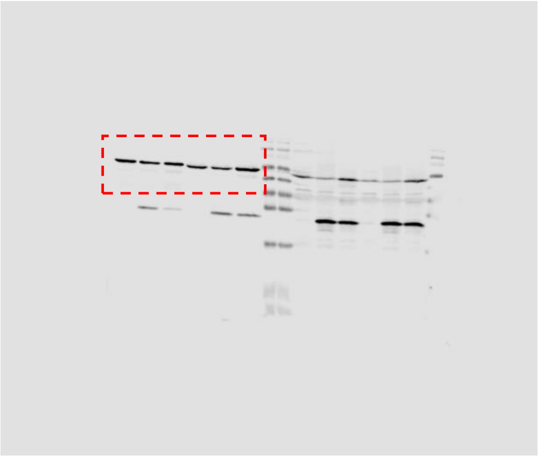

IP: TRIM21
IB: TRIM21

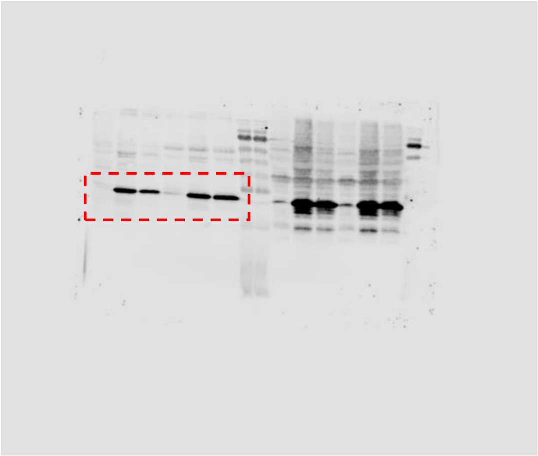

IP: TRIM21
IB: FLAG

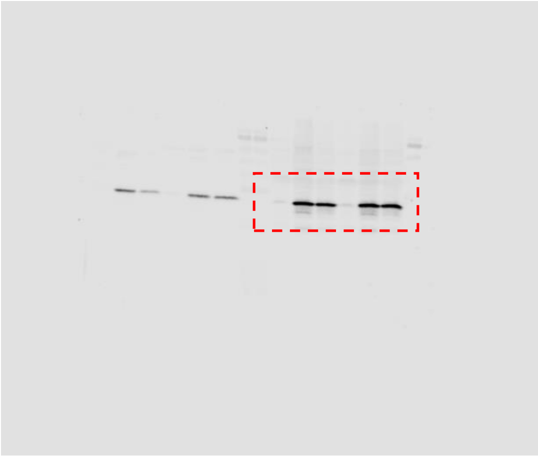

IB: FLAG
Load

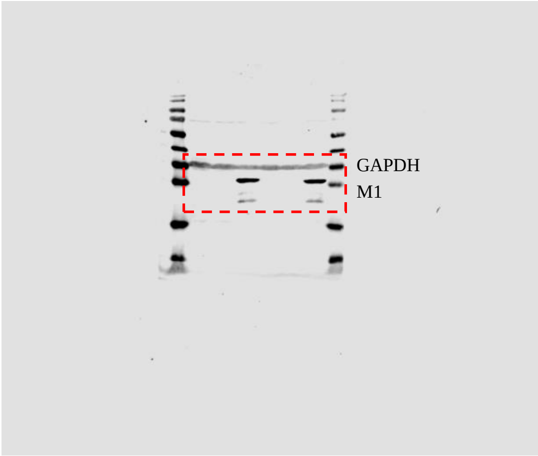

IB: M1 and GAPDH
Load

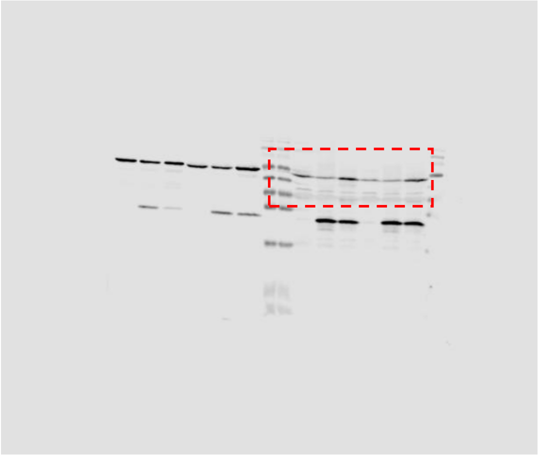

IB: TRIM21
Load

Figure S4A

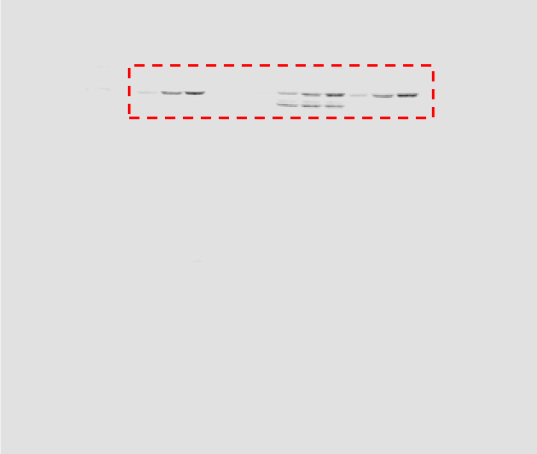

IB: TRIM21

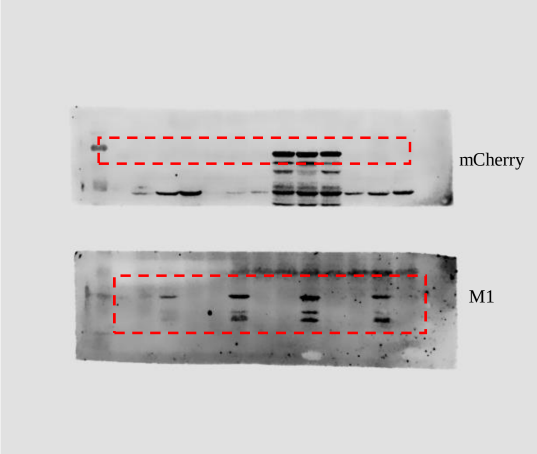

IB: M1 and mCherry

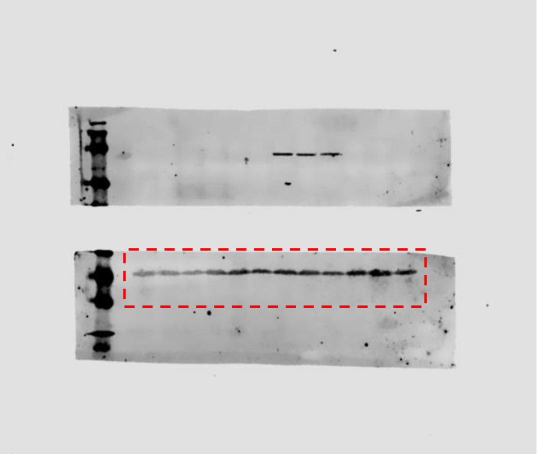

IB: GAPDH

Figure S4B

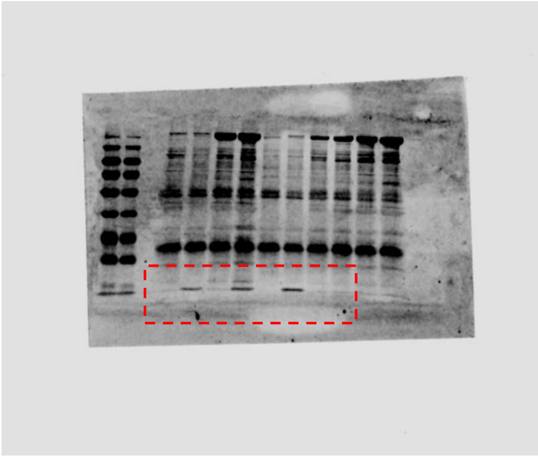

IP: FAT10
IB: FAT10

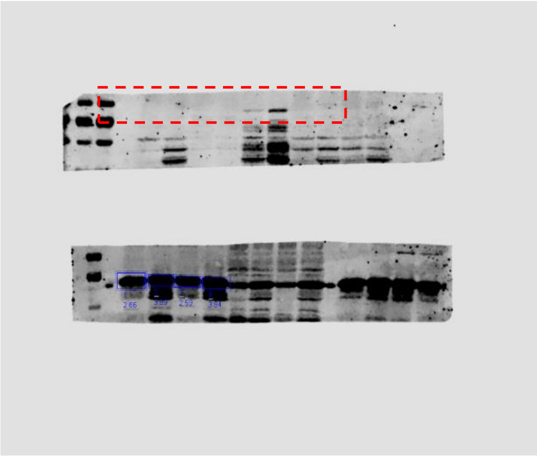

IP: mCherry
Load

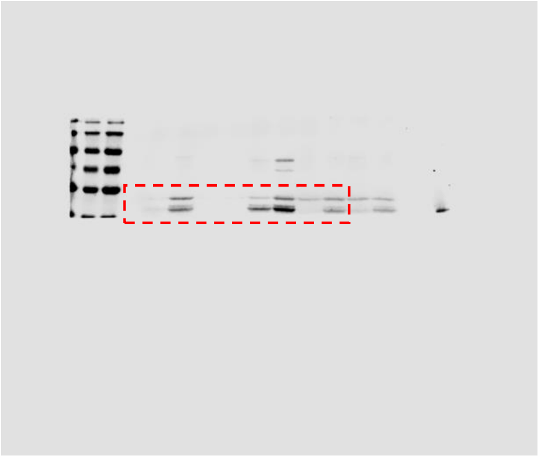

IP: TRIM21
Load

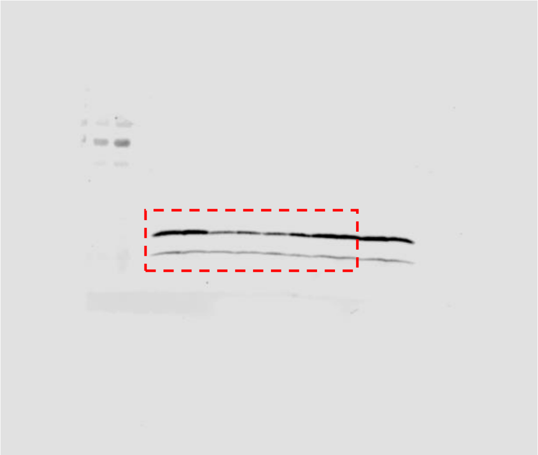

IP: GAPDH
Load

Figure S5B

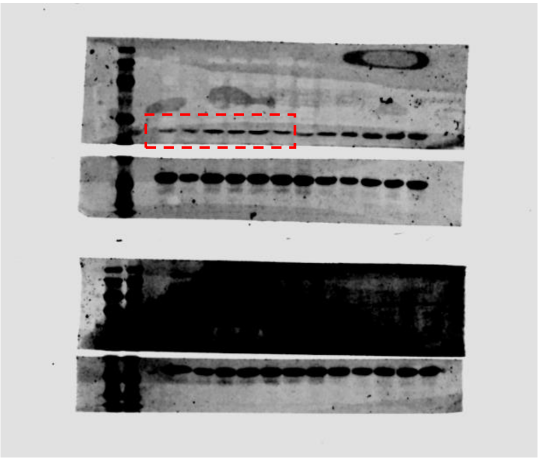

IB: TRIM21

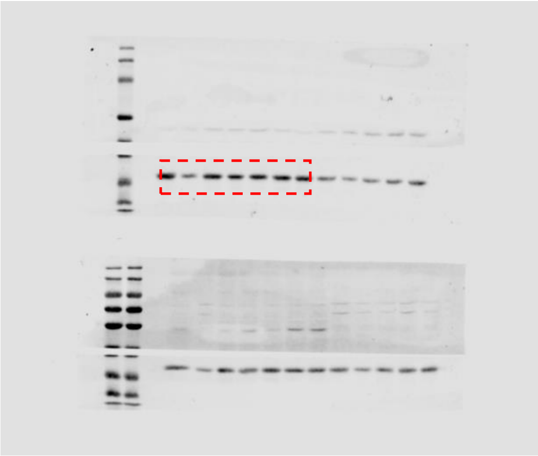

IB: GAPDH

Figure S5D

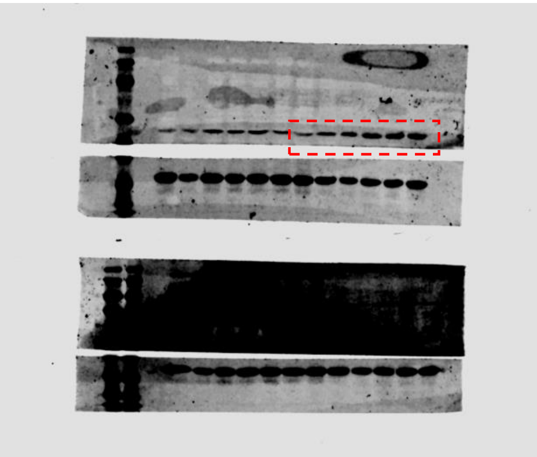

IB: TRIM21

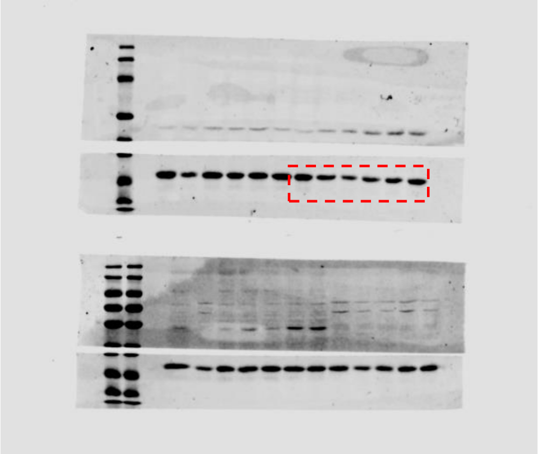

IB: GAPDH
